# The role of economic factors in shaping and constituting the household burden of neglected tropical diseases of the skin: Qualitative findings from Ghana and Ethiopia

**DOI:** 10.1016/j.socscimed.2024.117094

**Published:** 2024-09

**Authors:** Yohannes Hailemichael, Jacob Novignon, Lucy Owusu, Daniel Okyere, Tara Mtuy, Abebaw Yeshambel Alemu, Edmond Kwaku Ocloo, Eric Koka, Jennifer Palmer, Stephen L. Walker, Endalamaw Gadisa, Mirgissa Kaba, Catherine Pitt, SHARP Collaboration

**Affiliations:** a Armauer Hansen Research Institute, Addis Ababa, Ethiopia; b Kumasi Centre for Collaborative Research, Kwame Nkrumah University of Science & Technology, Kumasi, Ghana; c Department of Economics, Kwame Nkrumah University of Science & Technology, Kumasi, Ghana; d Noguchi Memorial Institute for Medical Research, University of Ghana, Accra, Ghana; e Faculty of Public Health and Policy, London School of Hygiene & Tropical Medicine, London, United Kingdom; f Department of Epidemiology and Biostatistics, Institute of Public Health, College of Medicine and Health Sciences, University of Gondar, Gondar, Ethiopia; g Department of Sociology and Anthropology, University of Cape Coast, Cape Coast, Ghana; h Faculty of Infectious and Tropical Diseases, London School of Hygiene & Tropical Medicine, London, United Kingdom; i School of Public Health, Addis Ababa University, Addis Ababa, Ethiopia

**Keywords:** Ghana, Ethiopia, Economic burden, Neglected tropical diseases, Household costs, Out-of-pocket, Insurance, Financial risk protection

## Abstract

Tracers of health system equity, neglected tropical diseases (NTDs) disproportionately affect marginalized populations. NTDs that manifest on the skin – “skin NTDs” – are associated with scarring, disfigurement, physical disability, social exclusion, psychological distress, and economic hardship. To support development and evaluation of appropriate intervention strategies, we aimed to improve understanding of the role of economic factors in shaping and constituting the burden that skin NTDs place on households. We collected data in 2021 in two predominantly rural districts: Atwima Mponua in Ghana (where Buruli ulcer, yaws, and leprosy are endemic) and Kalu in Ethiopia (where cutaneous leishmaniasis and leprosy are endemic). We conducted interviews (n = 50) and focus group discussions (n = 14) that explored economic themes with affected individuals, caregivers, and community members and analysed the data thematically using a pre-defined framework. We found remarkable commonalities across countries and diseases. We developed a conceptual framework which illustrates skin NTDs' negative economic impact, including financial costs of care-seeking and reductions in work and schooling; categorises coping strategies by their degree of risk-pooling; and clarifies the mechanisms through which skin NTDs disproportionately affect the poorest. Despite health insurance schemes in both countries, wide-ranging, often harmful coping strategies were reported. Traditional healers were often described as more accessible, affordable and offering more flexible payment terms than formal health services, except for Ethiopia's well-established leprosy programme. Our findings are important in informing strategies to mitigate the skin NTD burden and identifying key drivers of household costs to measure in future evaluations. To reduce skin NTDs' impact on households' physical, mental, and economic wellbeing, intervention strategies should address economic constraints to prompt and effective care-seeking. While financial support and incentives for referrals and promotion of insurance enrolment may mitigate some constraints, structural interventions that decentralise care may offer more equitable and sustainable access to skin NTD care.

## Highlights

• Neglected tropical diseases (NTDs) of the skin place an economic burden on households.
• Costs influence care-seeking choices and encourage use of traditional healers.
• Economic coping strategies used indicate inadequate financial risk protection.
• Our conceptual framework describes the role of economic factors in the skin NTD burden.
• Interventions must tackle economic factors inhibiting prompt and effective treatment.

## 1 Introduction

Neglected tropical diseases (NTDs) have been conceptualised as diseases affecting the world's poorest people and as tracers of equity in the path to Universal Health Coverage (UHC) (Fitzpatrick and Engels, 2015; Houweling et al., 2016; Molyneux et al., 2017). Skin NTDs – those NTDs with significant cutaneous manifestations – include leprosy, yaws, cutaneous leishmaniasis (CL), Buruli ulcer (BU), and several others; collectively, they remain important public health concerns in many countries (Mitra and Mawson, 2017; Yakupu et al., 2023). Neglected even amongst the NTDs, skin NTDs have received far less investment than helminth infections and other NTDs that can be addressed through mass administration of preventive chemotherapy (Smith et al., 2022). Skin NTDs are co-endemic in many communities and share some clinical features (Chandler and Fuller, 2018). Often chronic and requiring prolonged treatment, they are commonly associated with substantial physical disability, social exclusion, poor mental wellbeing, economic hardship, and reduced quality of life (Pescarini et al., 2018; Tilahun, 2014).

To improve intervention coverage and treatment outcomes, the World Health Organization (WHO) advocates integrated strategies to address multiple skin NTDs simultaneously (WHO, 2022). Ghana and Ethiopia have both developed national NTD master plans to improve diagnosis and treatment (FMoH, 2021; GHS, 2016); however, access to skin NTD services remains limited in both countries and elsewhere (Hotez et al., 2019; Molyneux et al., 2017; Semahegn et al., 2023). In Ghana, children under the age of 15 and people with poor access to water and sanitation have been reported to be disproportionately affected by yaws and BU (Okine et al., 2020). In Ethiopia, younger people are more at risk of CL, which is estimated to affect 20,000 to 50,000 persons per year (Alvar et al., 2012; Assefa, 2018), and new leprosy cases continue to emerge, with 3201 reported in 2019 (WHO, 2020).

A few economic analyses of the household burden of skin NTDs in the WHO Africa region have estimated the cost to households related to specific diseases, including, for example, BU in West Africa (Amoakoh and Aikins, 2013; Chukwu et al., 2017; Grietens et al., 2008) and leprosy in Nigeria (Ebenso et al., 2001) and Cameroon (Tembei et al., 2018). However, no studies have been done to date on the household costs of CL in Africa (Galvão et al., 2020; Sunyoto et al., 2019; Wijerathna et al., 2018). While generating some useful evidence of the substantial economic burden of individual skin NTDs, these cost analyses tend to focus narrowly on costs in a single context, are inconsistent in their inclusion of non-medical expenditure and productivity losses, and are not directly comparable. Moreover, these quantitative studies tend not to provide deeper insights into how and why economic burdens emerge. By contrast, many qualitative studies have explored household experiences of skin NTDs to understand these questions of ‘how’ and ‘why’ (Amoako et al., 2021; Eid et al., 2019; Nichter, 2019), but despite recognising the important role of economic factors, have tended to treat economic questions superficially and to focus on single diseases in single contexts.

We aim to improve understanding of the role of economic factors in shaping and constituting the burden of skin NTDs on households. We used qualitative methods to explore the costs of care-seeking, the additional costs to households of experiencing these diseases, and the strategies households adopt to cope with this economic burden. We also explored how economic factors shape care-seeking pathways and their potential to further impoverish individuals and their households. We collected data in Ghana and Ethiopia as part of multidisciplinary formative research to support the development of locally-adapted, integrated intervention strategies and the evaluation of their implementation (Kaba et al., 2024; Okyere et al., 2024). From our findings, we develop a conceptual framework, which illustrates the relationships between economic factors, context, care-seeking, and the burden of skin NTDs.

## 2 Methods

### 2.1 Study setting

Ghana and Ethiopia were selected for this study because they offer different cultural, epidemiological, and health systems contexts, while sharing substantial (though different) burdens of skin NTDs. To foster generalizability of findings, study districts were selected in both countries with relatively high skin NTD endemicity but without previous skin NTD interventions or research activities.

**Fig. 1:**
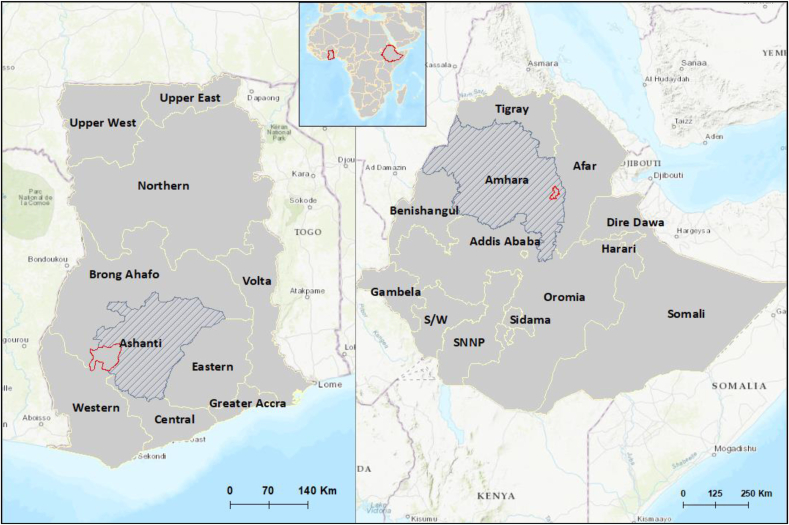
Map of study areas Map of Africa (inset, centre) shows Ghana and Ethiopia within Africa. Red outlines indicate the study areas: Atwima Mponua district in Ashanti Region in Ghana (left) and Kalu District in Amhara Region in Ethiopia (right). (For interpretation of the references to colour in this figure legend, the reader is referred to the Web version of this article.)

In Ghana, the study was conducted in Atwima Mponua District (population: 155,254) (GSS, 2021) in the Ashanti region (Fig. 1). The district has one district hospital, 9 health centres, and 6 community-based health planning and services (CHPS) facilities. The district is largely rural (84%), with about 12% of the region's population living below the national poverty line and a moderate income gap between the poor and rich (Gini coefficient: 38%) (GSS, 2017).

In Ethiopia, the study was conducted in Kalu District (or ‘woreda’) (population: 237,319 in 2020 (ESS, 2020)) in South Wollo Zone of Amhara Regional State (Fig. 1). The district has 9 health centres, 35 health posts, 20 private clinics, and 10 drug stores (KDHO, 2020). Since the conduct of this study, a primary hospital has been established. About 90% of the population reside in rural areas. Data on poverty and economic inequality levels are not available for the district; however, in 2016, 26% of Amhara region's population lived below the national poverty line (7184 birr ≈ $331 United States dollars per person per year (FDRoE, 2017; WB, 2016).

In both countries, the populations in the selected districts depend on subsistence farming and livestock-rearing, and small-scale trading is common. In Ghana, small-scale mining of natural resources, including gold, is widespread.

Healthcare in both countries is provided by traditional healers, government, and non-government health facilities (Kaba et al., 2024; Okyere et al., 2024). In Ethiopia, CL is often treated by traditional healers in the community. In the formal health service, CL treatment is provided at Boru Meda hospital, about 76 km from the farthest CL-affected community. Smaller CL lesions require multiple outpatient attendances for intralesional and/or cryotherapy, while more complex disease requires a minimum 28-day admission for systemic therapy. Since 2001, leprosy services have been integrated into primary health care services, allowing affected individuals to receive diagnosis and treatment with multi-drug therapy from a local health centre, with travel to Boru Meda only required for confirmation of the diagnosis. In Ghana, BU is sometimes treated by traditional healers. Wound care (usually necessary as part of BU treatment) is available at primary facilities, but diagnosis and medications were only available from the district capital, requiring multiple long journeys for some people.

Ghana's National Health Insurance Scheme (NHIS) covers about 35% of the region's population and more than 95% of health conditions (NHIA, 2018, 2023), while Ethiopia's Community Based Health Insurance (CBHI) reached about 60% coverage of the region population in 2020 (EHIA, 2020 unpublished). Both schemes are designed to cover all direct medical costs related to treating skin diseases, but individuals often have to pay for out-of-stock medications and supplies and for investigations not available at accredited health facilities and providers sometimes ask insured patients to pay for services officially covered by insurance (Agyepong and Nagai, 2011; Amporfu et al., 2023).

### 2.2 Data collection

The research team engaged with health workers, community leaders, and other stakeholders in the study district in Ghana in February 2021 and in Ethiopia in March 2021 to introduce the study objectives and planned activities and to initiate ongoing communication throughout the study and future intervention. Data were collected from February to June 2021, supervised by a field coordinator, a PhD student, and a post-doctoral research fellow in Ethiopia, and by senior social scientists and a health economist in Ghana.

Topic guides for individual interviews and focus group discussions (FGDs) were developed by the multidisciplinary, multi-country research team. They comprised open-ended questions on the economic impact of skin diseases, disease discourses, stigma, care-seeking pathways, health services capacity and readiness, and the policy landscape of skin NTDs. Participants in this multidisciplinary formative research were purposively sampled; they included individuals who had skin NTDs (currently or previously), caregivers, wider community members and leaders, traditional healers, healthcare workers, and policymakers. Themes explored were tailored to individual participants; for example, affected individuals and caregivers were asked about the actual costs they experienced, while community members were asked about their perceptions of costs and other factors, as such perceptions were hypothesised to influence care-seeking choices. In this article, we report findings from interviews and FGDs which included an exploration of economic themes from the perspectives of affected households and the wider community. In some cases, we include data from a health worker or teacher, where this person offered insights from the household or community perspectives.

We purposively sought affected individuals, caregivers, and community members (and healthcare workers) reflecting maximum variation in characteristics including age, sex, disease, locality (e.g. highland vs. lowland residence in Ethiopia), and care-seeking experience. We combined multiple techniques to identify participants meeting these criteria. First, two healthcare workers with experience caring for individuals with skin NTDs were interviewed at every health facility in each district. Affected individuals were identified from facility record review and discussion with healthcare workers, including community health workers in Ghana and disease focal persons and health extension workers in Ethiopia. Snowballing was then used to identify other affected individuals and caregivers in the community, including those unknown to the health service, who met purposive criteria. Selected community members were those who had some interaction with an individual with a skin NTD, whether as an extended family member, neighbour, friend, classmate, or colleague; they were identified through initial community engagement activities and through snowballing via affected households. In the context of the communities in which our research was conducted, healthcare workers lived in and were members of the wider community, and, because of the visibility of these diseases, affected individuals and their experiences tended to be fairly widely known beyond their own household.

Research assistants with qualifications in social science and public health and experience in data collection conducted interviews and FGDs in Amharic in Ethiopia and Twi in Ghana. In both countries, senior researchers trained the research assistants for one week on qualitative methods and data collection instruments. Interviews lasted a mean of 50 min (range: 15–90) and FGDs 60 min (range: 40–87). Research assistants took fieldnotes and recorded audio with consent.

### 2.3 Data management and analysis

Trained researchers transcribed interviews and FGDs verbatim and translated them into English. Transcriptions and translations were reviewed by MK, YH, EG, DO, and EKO for quality and consistency. All transcripts were uploaded into MAXQDA version 2020 (VERBI Software, 2021) for analysis.

**Fig. 2:**
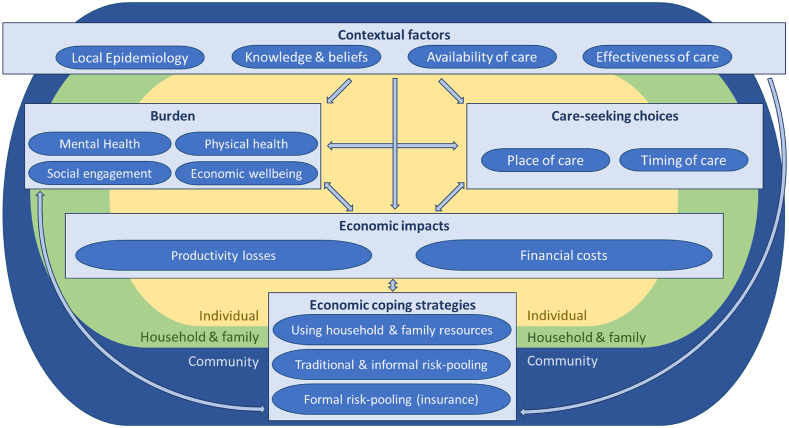
Conceptual framework: the relationships between economic factors, context, care-seeking, and the burden of skin NTDs.

The economic research leads (YH, CP, JN) developed a codebook, which combined pre-defined themes based on the research questions and additional themes that emerged from the data. All members of the research team agreed on the themes and subthemes in the codebook and on their relationships. Detailed coding and thematic analysis (both inductive and deductive) were done separately in each country by YH and JN. A conceptual framework was developed to guide further analysis and summarize the key concepts and their relationships that emerged, drawing on relevant economic and health behaviour theory (Fig. 2). In the results section, we first present the conceptual framework and then describe our detailed findings following this framework. Wherever possible, we attempt to draw out explicit comparisons between countries and diseases. Study methods are reported based on the Consolidated Criteria for Reporting Qualitative Research (COREQ) (Tong et al., 2007). When local currency values are reported, we also report United States dollars based on World Bank average exchange rates for 2021 ($1 = 5.81 GHS = 43.73 ETB) (WB, 2021).

### 2.4 Ethics

Ethical approval was received from the London School of Hygiene and Tropical Medicine, United Kingdom (22604); the Noguchi Memorial Institute for Medical Research, Ghana (022/20–21); and the Armauer Hansen Research Institute (PO/26/20/2021) and national ethics committee (722–506/m259), Ethiopia. Before initiation of an interview or FGD, a researcher explained the purpose of the study in detail to the participants in an appropriate language using an information sheet and obtained written informed consent or assent to participate in the study, to be audio recorded, to use anonymised quotations, and for the publication of the information obtained.

## 3 Results

We conducted and analysed 50 interviews (Table 1) and 14 FGDs (Table 2) that explored economic themes from a household perspective. Substantially more interviews (40 vs. 10) and FGDs (12 vs. 2) were conducted in Ethiopia than Ghana. Interview and FGD participants were aged 13–90 years old. Women comprised a majority of interview participants in Ghana (70%) and a minority (30%) in Ethiopia, and a minority of participants in FGDs in both countries (36%). Interviews were conducted with affected individuals (n = 41), caregivers (n = 6), local health workers (n = 2) and a teacher; FGDs were conducted exclusively with community members. Affected individuals and caregivers had experienced BU (n = 7), CL (n = 17), and leprosy (n = 23), although the CL cases were presumed while BU and Leprosy cases were confirmed.

**Table 1:** List of interview participants.

#	Unique ID	Skin NTD experienced	Sex	Age	Role
1	Ethiopia-IDI-060	CL	Female	14	Affected individual
2	Ethiopia-IDI-061	CL	Female	27	Affected individual
3	Ethiopia-IDI-053	CL	Female	40	Affected individual
4	Ethiopia-IDI-049	CL	Male	13	Affected individual
5	Ethiopia-IDI-070	CL	Male	17	Affected individual
6	Ethiopia-IDI-025	CL	Male	23	Affected individual
7	Ethiopia-IDI-028	CL	Male	23	Affected individual
8	Ethiopia-IDI-022	CL	Male	28	Affected individual
9	Ethiopia-IDI-051	CL	Male	30	Affected individual
10	Ethiopia-IDI-038	CL	Male	35	Affected individual
11	Ethiopia-IDI-026	CL	Male	39	Affected individual
12	Ethiopia-IDI-041	CL	Male	40	Affected individual
13	Ethiopia-IDI-043	CL	Male	40	Affected individual
14	Ethiopia-IDI-021	CL	Male	50	Affected individual
15	Ethiopia-IDI-023	CL	Male	58	Affected individual
16	Ethiopia-IDI-024	CL	Male	60	Affected individual
17	Ethiopia-IDI-030	CL	Female	40	Caregiver
18	Ethiopia-IDI-086	Leprosy	Female	40	Affected individual
19	Ethiopia-IDI-036	Leprosy	Female	50	Affected individual
20	Ethiopia-IDI-029	Leprosy	Female	60	Affected individual
21	Ethiopia-IDI-084	Leprosy	Female	60	Affected individual
22	Ethiopia-IDI-080	Leprosy	Female	70	Affected individual
23	Ethiopia-IDI-078	Leprosy	Male	45	Affected individual
24	Ethiopia-IDI-079	Leprosy	Male	90	Affected individual
25	Ethiopia-IDI-027	Leprosy	Male	23	Caregiver
26	Ethiopia-IDI-001	Leprosy	Female	35	Affected individual
27	Ethiopia-IDI-011	Leprosy	Female	28	Affected individual
28	Ethiopia-IDI-013	Leprosy	Female	50	Affected individual
29	Ethiopia-IDI-012	Leprosy	Male	28	Affected individual
30	Ethiopia-IDI-017	Leprosy	Male	40	Affected individual
31	Ethiopia-IDI-004	Leprosy	Male	43	Affected individual
32	Ethiopia-IDI-010	Leprosy	Male	48	Affected individual
33	Ethiopia-IDI-003	Leprosy	Male	52	Affected individual
34	Ethiopia-IDI-019	Leprosy	Male	55	Affected individual
35	Ethiopia-IDI-016	Leprosy	Male	61	Affected individual
36	Ethiopia-IDI-020	Leprosy	Male	65	Affected individual
37	Ethiopia-IDI-007	Leprosy	Male	71	Affected individual
38	Ethiopia-IDI-005	Leprosy	Male	75	Affected individual
39	Ethiopia-IDI-002	Leprosy	Male	35	Affected individual
40	Ethiopia-IDI-006	NA	Male	27	Teacher
41	Ghana-IDI-018	BU	Male	54	Affected individual
42	Ghana-IDI-010	BU	Female	45	Caregiver
43	Ghana-IDI-016	BU	Female	49	Caregiver
44	Ghana-IDI-008	BU	Female	42	Caregiver
45	Ghana-IDI-006	BU	Male	52	Caregiver
46	Ghana-IDI-023	BU	Female	39	Affected individual
47	Ghana-IDI-003	BU	Female	36	Affected individual
48	Ghana-IDI-021	Leprosy	Female	32	Affected individual
49	Ghana-IDI-057	NA	Female	38	Health worker
50	Ghana-IDI-029	NA	Male	27	Health worker

CL cases were presumed, while BU and leprosy cases were confirmed. CL: cutaneous leishmaniasis. BU: Buruli ulcer.

**Table 2:** List of focus group discussions (all held with community members).

#	Unique ID	Sex	Age range	Number of participants
1	Ethiopia-FGD-006	Female	18–20	8
2	Ethiopia-FGD-025	Female	20–45	7
3	Ethiopia-FGD-001	Female	25–37	7
4	Ethiopia-FGD-017	Female	25–56	8
5	Ethiopia-FGD-015	Male	18–20	8
6	Ethiopia-FGD-009	Male	18–37	9
7	Ethiopia-FGD-023	Male	22–34	7
8	Ethiopia-FGD-021	Male	27–32	6
9	Ethiopia-FGD-014	Male	29–39	6
10	Ethiopia-FGD-018	Male	35–42	7
11	Ethiopia-FGD-020	Male	40–65	8
12	Ethiopia-FGD-002	Male	45–60	6
13	Ghana-FGD-061	Female	18–25	6
14	Ghana-FGD-062	Male	35–62	6

Our conceptual framework (Fig. 2) illustrates the key themes and relationships that emerged from our findings. The framework indicates that economic factors are central to the skin NTD burden and care pathways used. We found that skin NTDs often had a negative economic impact on daily life in the form of both financial costs and productivity losses from care-seeking and from the burden of disease on daily lives. The negative impact fell predominantly on the affected individual and household and activated coping strategies at individual, household, and community levels, which offered varying degrees of risk-pooling and financial risk protection. Contextual factors, notably the local epidemiology, knowledge and beliefs, and effectiveness and availability of care for skin NTDs shaped the burden, care-seeking choices, and economic impacts of skin NTDs, as well as the coping strategies adopted. In the following sections, we describe the economic impact of skin NTDs on households in terms of the financial costs of care-seeking and productivity losses, and then the strategies households used to cope with this burden.

### 3.1 Economic impact of skin NTDs

#### 3.1.1 Financial costs of care-seeking

In both countries, individuals affected by skin NTDs reported substantial financial costs to obtain care, which were also perceived by the wider community. The major cost drivers in both countries were similar: transport between home and the care provider, medicines, medical supplies (syringes and gloves), and wound dressing material. A woman in Ghana explained the situation her daughter with BU faced:

*“She spends 120 cedis [$21] weekly for bandages and she buys drugs and this and that. The drug for the dressing runs out every three days.”* (Ghana-IDI-008, caregiver of a girl with BU)

In Ethiopia, multi-drug therapy (MDT) for leprosy had long been available free of charge; however, medicines for complications and comorbidities, medical supplies, and laboratory investigations were chargeable.

Costs were higher when patients were referred to facilities outside their community because they incurred higher travel costs. In Ethiopia, additional costs related to accommodation, investigations (electrocardiogram), laboratory tests, and food were reported for individuals with CL, who were typically treated as inpatients at Boru Meda hospital. A community member described his impression of the substantial costs associated with seeking care for CL:

*“When a person is infected with this disease [CL], even if he goes to the clinic or to traditional medicine, there are costs involved. If a person goes to Boru Meda hospital today for CL treatment, it could cost around 1000 birr [$23] to 3,000 birr [$69] just for medical treatment. In addition, the long hospital stay for 3 to 4 weeks costs much money.”* (Ethiopia-FGD-021, male participant)

These costs, in turn, affected care-seeking choices. A young woman explained how the costs of transport alone could inhibit treatment adherence, even when dire consequences were anticipated:

*“If the patient does not have money for transportation even though the treatment is free, the person would default treatment, which might worsen the disease to the extent that the person’s leg might be amputated.”* (Ghana-FGD-061, female participant)

In both countries, care from traditional healers was considered more affordable than care from formal providers because traditional healers lived locally and sometimes conducted home visits, which reduced transport costs, and because they offered flexible payment arrangements. Often described as an “appreciation,” this compensation could sometimes be determined by the affected individual and paid – whether in cash or in kind – when they are able and after they are healed. A man with BU in Ghana contrasted the impossibility of making immediate payments for hospital care with the flexibility offered by a traditional healer, and argued that cost was the primary motivation for preferring traditional care:

*“… But with the hospital, when they even prescribe a medicine for you, how do you go and buy them because you don’t have money. So, that’s why most people go there [traditional healer]. And with the 200 cedis [$34], they allowed time on it [delaying payment] until cocoa harvest. They know you don’t have [money] so they arrange the payment time with you.”* (Ghana-FGD-062, man with BU)

A young woman in Ethiopia similarly argued that “the only right place” to seek care was the hospital, but that cost motivated people to use traditional healers:

*“Boru Meda Hospital is the only right place to receive skin treatment but going to Boru requires money for transportation and other related expenses; overall, going to Boru is beyond the finances of many residents so they prefer to get treated here by the traditional healers.”* (Ethiopia-FGD-006, woman in school)

In Ethiopia, care-seeking from traditional healers was more prevalent among people with CL than leprosy. Individuals affected by leprosy reported a preference for formal care over traditional healers, which may reflect the longstanding decentralisation and integration of leprosy services.

#### 3.1.2 Productivity losses

Affected individuals and their household members experienced substantial productivity losses from care-seeking and the impact of skin NTDs on daily life.

##### 3.1.2.1 Productivity losses for individuals with skin NTDs

In both countries, the physical manifestation of skin NTDs - which included pain, discomfort, and loss of function – limited the ability of affected individuals to engage in their usual productive activities, whether school or work. The degree of productivity losses depended on the disease and its severity. A mother from Ghana described how her child could not attend school for months because of BU:

*“She stayed home for more than 2 months. She couldn’t go to school because when she did go to school, she would come back with a swollen leg and the wound would be bleeding.”* (Ghana-IDI-016, female caregiver of a girl with BU)

By contrast, a teenage boy in Ethiopia described missing only a few days of school because of his CL:

*“When I get sick [with CL], I cannot go to school. I was absent for about four days. But I didn’t entirely drop out.”* (Ethiopia-IDI-049, man with CL)

In Ghana, a man described how pain from BU prevented him from taking paid employment:

*“Some time ago, [name of mining company] came and they needed security persons. I wrote an application, I was called for an interview, but the boots they gave me, I couldn't wear them because of my leg so I stopped.”* (Ghana-IDI-018, man with BU)

Similarly, in Ethiopia, a man described how the physical impact of leprosy prevented him from farming:

*“Previously, I used to plough and produce. I was a strong farmer. After I got this disease [leprosy], I stopped farming. I’m unable to plough, produce, and breed cattle.”* (Ethiopia-IDI-007, man with leprosy)

In Ethiopia, traditional beliefs about skin conditions and their treatment also contributed to productivity losses, even in circumstances where individuals were physically able to undertake work. For example, most of those interviewed with leprosy believed agricultural activities worsened the disease and thus avoided farming, as one man explained:

*“… … I don’t touch the soil. When I touch the soil, I feel as if something gets in my body and I get sick. The disease [leprosy] hates farming*.*”* (Ethiopia-IDI-001, man with leprosy)

The effectiveness of traditional treatment for CL was believed to decrease if affected individuals interacted with other members of the community. Traditional healers therefore encouraged their patients to stay home alone with windows and doors closed to avoid interacting with others, who may inadvertently cast a “bad spirit” or “shadows” (in Amharic, “*tila*”) on them. People who were menstruating, had had sex the previous night, or were affected by other circumstances associated with impurity were described as casting shadows. A man with CL explained this widely held belief:

*“When I apply the ointment, I can’t move around and interact with people for fear of the shadows*. *The shadow will fall on you and the infection will get worse.”* (Ethiopia-IDI-041, man with CL)

##### 3.1.2.2 Productivity losses for household members

In both countries, skin NTDs also reduced the productivity of other household members, who accompanied the affected individual in care-seeking and provided care and support at home. In Ethiopia, men were generally expected to accompany household members (both women and children) to seek treatment. A woman with CL, for example, described how her husband accompanied her to seek treatment:

*“ …. we went twice for treatment and he [husband] lost two days of farming activity.”* (Ethiopia-IDI-053, woman with CL)

In Ghana, however, women were more likely to forgo their economic activities both to accompany the affected individual to seek treatment and to provide care, while men were expected to work to afford the cost of treatment. One woman thought to have BU recounted how her mother was no longer able to earn money because she stayed home to care for her:

*“She [mother] sells sachet water in a secondary school. She used to go to work from Monday to Friday but because of me she doesn’t go anymore.”* (Ghana-IDI-003, woman presumed with BU)

In Ethiopia, carers also described loss of education, which was not observed in Ghana:

*“… I had to go to the market and then to school. Every responsibility was put on me. Finally, I dropped out of school when I became busy with work and caring for my mother.”* (Ethiopia-IDI-027, male caregiver of a woman with leprosy)

### 3.2 Household strategies for coping with the economic burden of skin NTDs

Across the two countries, coping strategies ranged from formal, informal, and traditional risk-pooling arrangements to use of individual and household resources.

#### 3.2.1 Formal risk-pooling arrangements

Formal insurance schemes allowed a degree of risk-pooling in both countries, but participants described important gaps in coverage. In Ghana, the NHIS covered direct medical costs for active subscribers, while non-subscribers paid out of pocket. Two women described the charges they faced because they lacked an active subscription:

*“I have health insurance, but it had expired. Even the first day I went, they told us to pay 400 cedis [$69] before they started treatment.”* (Ghana-IDI-023, woman formerly with BU)

*“I did not have an insurance card when I visited the hospital, so I incurred a cost of 500 cedis [$86]*.” (Ghana-IDI-021, woman with leprosy)

In Ethiopia, CBHI was described as providing valuable benefits for individuals with skin NTDs. A woman described how the scheme covered her leprosy treatment costs:

*“Before the start of health insurance, I used to cover the cost of my care. Nowadays, community-based health insurance covers my medical cost. The services were also free of charge except the transportation cost.”* (Ethiopia-IDI-084, woman with leprosy)

However, in both countries, stockouts of medications and other treatment supplies resulted in individuals paying out-of-pocket for services notionally covered in the benefits package. A health worker in Ghana explained that prescribed medications may not all be covered by the scheme:

*“Patients who are not registered with NHIS pay for their healthcare and medications. It is not all medications prescribed by physicians that are covered by NHIS. So, even if a patient is registered with NHIS, the patient would have to pay for the medication which is not covered by NHIS.”* (Ghana-IDI-029, male health worker)

In Ethiopia, a teacher reported that people do not always receive all the services at health facilities that are notionally covered by CBHI:

*“I observe health insurance often has a lot of challenges. Sometimes the community complains that they do not get service despite having the health insurance.”* (Ethiopia-IDI-006, male teacher)

#### 3.2.2 Informal and traditional risk-pooling arrangements

In both countries, additional community-level risk-pooling arrangements also provided support of varying extent and form. This support tended to be described more by community members than by the affected individuals themselves, and there were indications of gaps and potential inequities in coverage and often expectations that support would be repaid.

In Ghana, community members sometimes made informal contributions in kind or cash directly to the affected household. A man explained that he and his wife, who had BU, benefited from generous community donations which he indirectly attributed to his social standing:

*“People used to come and visit her. My wife and I are well known by a lot of people and we are church leaders as well. So, when it happened, a lot of people came to visit her, and gave us their full support either in cash or kind. So, we didn’t really have any financial challenges.”* (Ghana-IDI-006, male caregiver of a woman with BU)

A health worker noted that community health committees sometimes pooled resources from the wider community to support treatment costs of affected individuals in need. However, this practice occurred inconsistently on an ad hoc basis, as the committee has no mandate to distribute resources in this way.

*“We have community health committees who sometimes support patients financially when the need arises. For example, if the patient needs to travel to the district hospital but does not have the means of transport, the community health committee intervenes for the person to get to the facility. Sometimes, too, when patients are admitted at the hospital and can't pay for the bills and other medications, the committee also assists to pay so that the person receives care and then after the person is better, he pays the money back.”* (Ghana-IDI-057, female health worker)

In Ethiopia, participants described several traditional forms of community risk-pooling. For example, a young man explained how community members supported farming or harvesting for those in need due to poor health:

*“When a person is sick, whether it is a skin disease or another that makes him bed-ridden, and if that person has work on the farm, the work is covered by the communal labour. We call it ‘debo’ [communal labour].”* (Ethiopia-FGD-009, young male community member)

Another type of community support involved traditional risk pooling groups called “*edir*.” Members of these groups received pre-agreed amounts when they experienced social, economic or health problems, as one community member described:

*“If someone is sick and short of money, the person will receive some money from edir. The person can take adequate money and travel. After the person recovers, there is a kind of obligation and agreement that the person can return the money over time or be exempted*.*”* (Ethiopia-FGD-001, female community member)

#### 3.2.3 Coping strategies within the household and wider family

In both countries, reported coping strategies included relying on other family members, dis-saving (i.e. spending savings), and finding less physically demanding jobs. In Ethiopia, the sale of assets, reduced consumption, borrowing, and contracting out farmlands were also described. While some households appeared to cope well, others described serious hardship and difficult choices or employing coping strategies that reduced their household's long-term economic security. These hardships indicated that neither the formal nor informal risk-pooling strategies offered complete financial risk protection for all households affected by skin NTDs.

Some affected individuals described receiving adequate support from within their families to cover treatment costs and being able to cope well; one woman with 10.13039/100008473BU, for example, described feeling well-supported by her immediate family:

*“We didn’t face any challenge here because my husband and two children are all working so they supported me. So, I didn’t see my condition bringing any financial challenge.”* (Ghana-IDI-023, woman with BU)

Use of savings, whether of the affected individual or their wider household, was often described. Speaking about a woman with BU, one caregiver explained that her savings had been intended for a building:

*“The disease has drained all the money she saved and was using in her building project in her hometown.”* (Ghana-IDI-008, female caregiver)

Similarly, a woman described how she used her husband's savings for her own leprosy treatment:

*“I just paid for health care once and twice from what he [husband] gave me of the money saved.”* (Ethiopia-IDI-001, woman with leprosy)

In both countries, men affected by leprosy or BU described needing to seek alternative, less physically demanding work, such as business or looking after grazing livestock (rather than more strenuous aspects of farming). One man with leprosy who was able to find a new, less physically demanding job described how it made him feel “like an old man”:

*“… Yes, definitely, it [leprosy] has livelihood impact; my land was not ploughed for food. My life was hand to mouth, and it forces me to drop all farming activities. Thanks to God, I am leading my life by working on this grain-milling machine like an old man.”* (Ethiopia-IDI-016, man with leprosy)

Similarly, a man in his 60s with a BU affecting his leg explained that he needed to find a job that would not require him to stand:

*“The only work I think I can do is the one that requires me to sit at a particular place - anything that involves my hands without the use of my legs, like cracking cocoa pods open.”* (Ghana-IDI-002, man with BU)

In Ethiopia, a wide variety of additional coping strategies were reported, which participants in Ghana did not mention. Sale of assets, for example, was reported as a means to raise cash to pay for upfront treatment costs. Despite the decentralisation of leprosy care and availability of free MDT, a man recounted selling his ox to pay for travel, food, accommodation, and laboratory tests for his leprosy treatment:

*“I sold my ox for 9000 birr [$206] to get treatment. That money was finished when we had made the journeys back and forth. Now, I don’t have anything.”* (Ethiopia-IDI-005, man with leprosy)

Families affected by leprosy and CL reported reducing their food consumption to raise money for treatment, as one man explained in dramatic terms:

*“I don’t know whether I die today or tomorrow, so I tend to decrease the little that I eat at home.”* (Ethiopia-IDI-007, man with leprosy)

Borrowing from other members of the community was also prevalent. A man with CL described how he “got a loan from another person when [he] went there [hospital] for treatment,” (Ethiopia IDI-011) while a man with leprosy, who described himself as “poor,” mentioned borrowing money from a neighbour to pay for treatment (Ethiopia IDI-005). To cope with being unable to farm, participants reported leasing their farmland. A man who stopped farming because of his leprosy recounted how leasing his farm meant he now only received half of what he would have received if he had been able to farm himself:

*“I just lease my land with a farmer. The person will take half of what was produced, and I will take the remaining.”* (Ethiopia-IDI-002, man with leprosy)

## 4 Discussion

Our qualitative study has elucidated the important role of economic factors in the substantial burden that skin NTDs place on households, particularly the most vulnerable, which we have illustrated in a conceptual framework. By exploring the experiences of households affected by skin NTDs through an economic lens, we have shown how economic factors shape care-seeking choices, further exacerbating their economic impact. We have shown that skin NTDs place a substantial economic burden on households in the form of financial costs of care-seeking and missed work and schooling for both affected individuals and their household members. We have also categorised coping strategies according to their degree of risk-pooling and highlighted how some household and family-level coping strategies risked weakening long-term economic security and indicated gaps in financial risk protection afforded by formal and informal risk-pooling mechanisms. Our findings are important in clarifying the mechanisms through which skin NTDs may disproportionately affect the poorest households, informing strategies to mitigate this burden, and identifying key drivers of household costs to assess in future evaluations.

We identified remarkable commonalities between the two very different contexts in which our study was undertaken. For example, notwithstanding the many factors that may motivate care-seeking from different kinds of traditional healers (which we explore elsewhere [Kaba et al., 2023; Okyere et al., 2023]), we found that economic rationales featured prominently in both countries, as traditional healers were described as more accessible (involving lower travel costs), more flexible in their payment terms (accepting “appreciations” over time, rather than substantial upfront payments), and were sometimes perceived as less expensive overall. In Ethiopia, however, leprosy-affected individuals tended to seek care from the health service, reflecting the longstanding integration and decentralisation of leprosy services into the health service, though they nonetheless sometimes sold assets and reduced food consumption to afford treatment and travel. Across diseases, common factors included the fact that the diseases manifest on the skin and become apparent to the affected individuals to prompt care seeking; become progressively worse over time without effective treatment and may be associated with stigma; require specific expertise to diagnose and treat effectively, which is often only available at a distant hospital; and consequently, impose both financial costs and productivity losses on households, which varied across individuals. Our findings suggested that direct costs of care-seeking tended to be higher for CL when hospitalisation and additional tests were required; productivity losses associated with care-seeking appeared higher for BU when multiple wound dressing changes were required; and both BU and leprosy could incur lifelong productivity losses when they resulted in permanent physical disabilities.

The cross-country comparative approach of our study has allowed us to elucidate commonalities in the experiences of people affected by skin NTDs, while also recognising specificities associated with disease and contextual characteristics. While we conducted our analysis through an economic lens, our multidisciplinary team allowed us to situate our findings within a wider context of factors shaping households’ experiences of skin NTDs. We successfully included a valuable range of perspectives, including those of affected individuals, their caregivers, and wider community members; however, our work also has limitations. Locating a diverse range of affected individuals was challenging in both countries. Our interviews with affected individuals and their caregivers in Ghana mainly focused on BU, while Ethiopia focused on CL and, to a lesser extent, leprosy, and we were able to include relatively fewer affected individuals in Ghana. In both countries, to reflect a range of treatment experiences, our sample of affected individuals included both those who had obtained a formal diagnosis and those whose diagnoses were presumed. We identified gendered norms in caregiving (which disproportionately imposed opportunity costs on women) and in notions of appropriate accompaniers for care-seeking (which differed between countries); however, we were unable to explore gendered differences in intra-household resource allocation, decision-making or the costs of care, which have been described elsewhere (Nanda, 2002; Witter et al., 2017). Consistent with previous research (Stienstra et al., 2002; Tuwor et al., 2024) our wider formative studies found that BU, CL, and leprosy could all decrease attractiveness and ability to marry or remain married for men and especially women (Kaba et al., 2024; Okyere et al., 2024); however, we were unable to explore whether these social difficulties had economic consequences for affected individuals.

Responding to calls for greater use of qualitative methods in NTD research (Allotey et al., 2010) and in health economics (Coast and De Allegri, 2018; Coast, 1999; Smith et al., 2009), we have explored household experiences and choices around skin NTDs using an economic lens. We have shown that decision-making around care-seeking for skin NTDs is not motivated exclusively by knowledge and beliefs; resources play an important role, too. While economic factors are often cited in passing in qualitative studies of NTDs (Bautista-Gomez et al., 2022; Semahegn et al., 2023; van Wijk et al., 2021; Velink et al., 2016) and in analyses of their disease burden (Mitra and Mawson, 2017; Yakupu et al., 2023), our explicit economic focus has allowed us to identify the mechanisms by which NTDs may most affect the poorest and exacerbate inequities. Our approach of looking across multiple skin NTDs is important for informing current policy efforts towards integrated approaches that address the burden of multiple NTDs simultaneously while accounting for their commonalities and differences (WHO, 2020).

Our finding that the insurance schemes in both countries left substantial gaps in financial risk protection for those affected by skin NTDs echoes evidence from many African countries of shortcomings in formal health financing arrangements, notably insurance schemes and CBHI in particular (Barasa et al., 2021; Ly et al., 2022). While we found evidence that a variety of informal risk-pooling arrangements in Ghana and traditional arrangements in Ethiopia (Mariam, 2003; Ololo et al., 2009) mitigated some of the economic impact of skin NTDs for some people, this community support seemed more available to households with stronger social connections, as has been noted previously (McIntyre, 2006). To fill the gaps left by insurance and community support, households and wider families used a diverse range of strategies to cope with out-of-pocket payments. These strategies drew on very different pre-existing levels of household savings and social networks, and many – particularly, the sale of assets, school withdrawal, some changes of occupation, and borrowing – risked damaging a household's long-term economic wellbeing (Sparrow et al., 2015; Van Damme et al., 2004).

The conceptual framework that emerged from our findings builds on a seminal conceptualisation of the economic burden of illness on households (McIntyre, 2006) most notably by delineating the feedback loops by which the economic impact of disease influences care-seeking choices and thus health outcomes, as well as the important role of context. We also build on conceptualisations of skin NTDs, which focus on mental health and stigma, but omit economic questions (Koschorke et al., 2022; Pires et al., 2019). Drawing on the socio-ecological model of health behaviour, we situate individuals’ care-seeking choices within their household and family and wider community contexts and recognise the interplay between these levels of influence; the implication is that effective strategies to shape care-seeking behaviour must target multiple levels (Sallis et al., 2008). Our conceptual framework can be used to analyse the economic burden of skin NTDs, to inform the design of intervention strategies, and potentially to elucidate the household experience of other chronic conditions. Further research is needed to explore the experiences of skin NTDs in other contexts, including amongst refugees and conflict-affected populations; the equity aspects of experiencing skin NTDs; and the usefulness of our conceptual framework.

Understanding household cost drivers is important both for designing future economic evaluations and for designing interventions themselves. Whereas previous “economic” interventions for skin NTDs have tended to focus on microfinance and employment opportunities for those facing social exclusion and long-term disability from leprosy or other skin NTDs (Dadun et al., 2019; van ’t Noordende et al., 2022), we have shown how an economic lens can be used to identify the important material factors and incentives that contribute to care-seeking choices. By facilitating earlier access to effective care, interventions that address these economic factors have the potential to improve treatment outcomes, reduce physical and mental health consequences (Amoussouhoui et al., 2018; Van Veen et al., 2006), and thus reduce the need for long-term economic and other support. Such interventions could include, for example, financing patient transportation costs to centralised treatment centres, which have been shown to improve care-seeking and treatment adherence for BU (Ahorlu et al. (2013). Broad health system improvements, notably in the effectiveness and coverage of risk-pooling arrangements and in the availability of necessary medicines and supplies for skin NTD care, would address some of the supply challenges raised. However, decentralising service provision for skin NTDs into primary care could offer a more sustainable, accessible, and equitable solution.

## Membership of SHARP collaboration

Dorothy Yeboah-Manu, Michael Marks, Rachel Pullan, Richard O. Phillips, Saba Lambert, Yaw Amoako, Collins Ahorlu, Ruth Tuwor, Teklu Cherkose, Adwoa Asante-Poku, Esther Amon, Ishaque Mintah Siam, Emmanuel Kyei Afreh, Abigail Agbanyo.

## Funding statement

The Skin Health Africa Research Programme (SHARP) is a collaboration between the London School of Hygiene and Tropical Medicine in the UK, the Noguchi Memorial Institute for Medical Research and the Kumasi Centre for Collaborative Research in Tropical Medicine of Kwame Nkrumah University of Science and Technology in Ghana, and the Armauer Hansen Research Institute and Addis Ababa University in Ethiopia. SHARP is funded by the United Kingdom's 10.13039/501100000272National Institute for Health and Care Research through the Research and Innovation for Global Health Transformation programme (Reference NIHR200125). The funders had no role in study design, data collection and analysis, decision to publish, or preparation of the manuscript.

## CRediT authorship contribution statement

**Yohannes Hailemichael:** Writing – original draft, Supervision, Investigation, Formal analysis, Data curation, Writing – review & editing. **Jacob Novignon:** Conceptualization, Formal analysis, Visualization, Writing – original draft, Writing – review & editing. **Lucy Owusu:** Investigation, Writing – review & editing. **Daniel Okyere:** Data curation, Investigation, Writing – review & editing. **Tara Mtuy:** Validation, Writing – review & editing. **Abebaw Yeshambel Alemu:** Writing – review & editing, Investigation, Data curation. **Edmond Kwaku Ocloo:** Writing – review & editing, Investigation, Data curation. **Eric Koka:** Writing – review & editing. **Jennifer Palmer:** Writing – review & editing, Validation. **Stephen L. Walker:** Writing – review & editing, Funding acquisition. **Endalamaw Gadisa:** Writing – review & editing, Funding acquisition. **Mirgissa Kaba:** Writing – review & editing, Methodology, Funding acquisition. **Catherine Pitt:** Writing – review & editing, Writing – original draft, Visualization, Methodology, Funding acquisition, Formal analysis, Conceptualization.

## Data Availability

Data will be made available on request.

## References

[bib1] Agyepong I.A., Nagai R.A. (2011). We charge them; otherwise we cannot run the hospital” front line workers, clients and health financing policy implementation gaps in Ghana. Health Pol..

[bib2] Ahorlu C.K. (2013). Enhancing Buruli ulcer control in Ghana through social interventions: a case study from the Obom sub-district. BMC Publ. Health.

[bib3] Allotey P. (2010). Social sciences research in neglected tropical diseases 1: the ongoing neglect in the neglected tropical diseases. Health Res. Pol. Syst..

[bib4] Alvar J. (2012). Leishmaniasis worldwide and global estimates of its incidence. PLoS One.

[bib5] Amoako Y.A. (2021). Caregiver burden in buruli ulcer disease: evidence from Ghana. PLoS Neglected Trop. Dis..

[bib6] Amoakoh H.B., Aikins M. (2013). Household cost of out-patient treatment of Buruli ulcer in Ghana: a case study of Obom in Ga South Municipality. BMC Health Serv. Res..

[bib7] Amoussouhoui A.S. (2018). Implementation of a decentralized community-based treatment program to improve the management of Buruli ulcer in the Ouinhi district of Benin, West Africa. PLoS Neglected Trop. Dis..

[bib8] Amporfu E. (2023). Billing the insured: an assessment of out-of-pocket payment by insured patients in Ghana. Health Serv. Insights.

[bib9] Assefa A. (2018). Leishmaniasis in Ethiopia : a systematic review and meta-analysis of prevalence in animals and humans. Heliyon.

[bib10] Barasa E. (2021). Examining the level and inequality in health insurance coverage in 36 sub-Saharan African countries. BMJ Glob. Health.

[bib11] Bautista-Gomez M.M. (2022). Barriers to cutaneous leishmaniasis care faced by indigenous communities of rural areas in Colombia: a qualitative study. BMC Infect. Dis..

[bib12] Chandler D.J., Fuller L.C. (2018). The skin—a common pathway for integrating diagnosis and management of NTDs. Tropical Medicine and Infectious Disease.

[bib13] Chukwu J.N. (2017). Financial burden of health care for Buruli ulcer patients in Nigeria: the patients' perspective. International Health.

[bib14] Coast J. (1999). The appropriate uses of qualitative methods in health economics. Health Econ..

[bib15] Coast J., De Allegri M. (2018). Oxford Research Encyclopedia : Economics and Finance.

[bib16] Dadun D. (2019). Assessing the impact of the twin track socio-economic intervention on reducing leprosy-related stigma in Cirebon District, Indonesia. Int. J. Environ. Res. Publ. Health.

[bib17] Ebenso B.E. (2001). Treatment outcome and impact of leprosy elimination campaign in Sokoto and Zamfara states, Nigeria. Lepr. Rev..

[bib18] Eid D. (2019). Leishmaniasis patients' pilgrimage to access health care in rural Bolivia: a qualitative study using human rights to health approach. BMC Int. Health Hum. Right.

[bib19] ESS (2020). Ethiopian Statistical Service, Addia Ababa.

[bib20] FDRoE (2017).

[bib21] Fitzpatrick C., Engels D. (2015). Leaving no one behind: a neglected tropical disease indicator and tracers for the Sustainable Development Goals. International Health.

[bib22] FMoH (2021).

[bib23] Galvão E.L. (2020). Economic impact of localized cutaneous leishmaniasis on adult patients of a referral service in Belo Horizonte, Minas Gerais State, Brazil. Cad. Saúde Pública.

[bib24] GHS (2016).

[bib25] Grietens K.P. (2008). “It is me who endures but my family that suffers”: social isolation as a consequence of the household cost burden of buruli ulcer free of charge hospital treatment. PLoS Neglected Trop. Dis..

[bib26] GSS (2017).

[bib27] GSS (2021).

[bib28] Hotez P.J. (2019). Ghana: accelerating neglected tropical disease control in a setting of economic development. PLoS Neglected Trop. Dis..

[bib29] Houweling T.A.J. (2016). Socioeconomic inequalities in neglected tropical diseases: a systematic review. PLoS Neglected Trop. Dis..

[bib30] Kaba M. (2024). Understanding experiences of neglected tropical diseases of the skin: a mixed-methods study to inform intervention development in Ethiopia. Under Review.

[bib31] KDHO (2020).

[bib32] Koschorke M. (2022). Mental health, stigma, and neglected tropical diseases: a review and systematic mapping of the evidence. Frontiers in Tropical Diseases.

[bib33] Ly M.S. (2022). Universal health insurance in Africa: a narrative review of the literature on institutional models. BMJ Glob. Health.

[bib34] Mariam D.H. (2003). Indigenous social insurance as an alternative financing mechanism for health care in Ethiopia (the case of eders). Soc. Sci. Med..

[bib35] McIntyre D. (2006).

[bib36] Mitra A.K., Mawson A.R. (2017). Neglected tropical diseases: epidemiology and global burden. Tropical Medicine and Infectious Disease.

[bib37] Molyneux D.H. (2017). Neglected tropical diseases: progress towards addressing the chronic pandemic. Lancet.

[bib38] Nanda P. (2002). Gender dimensions of user fees: implications for women's utilization of health care. Reprod. Health Matters.

[bib39] NHIA (2018).

[bib40] NHIA (2023).

[bib41] Nichter M. (2019). Social science contributions to BU focused health service research in West-Africa. Buruli Ulcer: Mycobacterium Ulcerans Disease.

[bib42] Okine R.N.A. (2020). Factors associated with cutaneous ulcers among children in two yaws-endemic districts in Ghana. Infectious Diseases of Poverty.

[bib43] Okyere D., Ocloo E.K., Owusu L., Amoako Y.A., Tuwor R.D., Koka E., Novignon J., Asante-Poku A., Siam I.M., Afreh E.K., Agbanyo A. (2024). Improving experiences of neglected tropical diseases of the skin: Mixed methods formative research for development of a complex intervention in Atwima Mponua District, Ghana. PLOS Glob. Public Health.

[bib44] Ololo S. (2009). Indigenous community insurance (iddirs) as an alternative health care financing in jimma city, southwest Ethiopia. Ethiopian Journal of Health Sciences.

[bib45] Pescarini J.M. (2018). Socioeconomic risk markers of leprosy in high-burden countries: a systematic review and meta-analysis. PLoS Neglected Trop. Dis..

[bib46] Pires M. (2019). The impact of leishmaniasis on mental health and psychosocial well-being: a systematic review. PLoS One.

[bib47] Sallis J.F., Glanz K., Viswanath K. (2008). Health Behaviour and Health Education: Theory, Research and Practice.

[bib48] Semahegn A. (2023). Burden of neglected tropical diseases and access to medicine and diagnostics in Ethiopia : a scoping review. Syst. Rev..

[bib49] Smith J.S. (2022). Financing care for severe stigmatizing skin diseases (SSSDs) in Liberia: challenges and opportunities. Int. J. Equity Health.

[bib50] Smith N. (2009). Qualitative methods in health-care priority setting research. Health Econ..

[bib51] Sparrow R. (2015). Coping with the economic consequences of ILL health in Indonesia. Health Econ..

[bib52] Stienstra Y. (2002). Beliefs and attitudes toward buruli ulcer in Ghana. Am. J. Trop. Med. Hyg..

[bib53] Sunyoto T. (2019). Understanding the economic impact of leishmaniasis on households in endemic countries: a systematic review. Expert Review of Anti-Infective Therapy.

[bib54] Tembei A.M. (2018). A comparative analysis of economic cost of podoconiosis and leprosy on affected households in the northwest region of Cameroon. Am. J. Trop. Med. Hyg..

[bib55] Tilahun F. (2014). Magnitude and associated factors of cutaneous leishmaniasis; in mekelle city, ayder referral hospital, tigray, northern Ethiopia, 2014. Clin. Med. Res..

[bib56] Tong A. (2007). Consolidated criteria for reporting qualitative research (COREQ): a 32-item checklist for interviews and focus groups. Int. J. Qual. Health Care.

[bib57] Tuwor R. (2024).

[bib58] van ’t Noordende A.T. (2022). Family-based intervention for prevention and self-management of disabilities due to leprosy, podoconiosis and lymphatic filariasis versus usual care in Ethiopia: study protocol for a cluster-randomised controlled trial. BMJ Open.

[bib59] Van Damme W. (2004). Out-of-pocket health expenditure and debt in poor households: evidence from Cambodia. Trop. Med. Int. Health.

[bib60] Van Veen N.H.J. (2006). The relationship between detection delay and impairment in leprosy control: a comparison of patient cohorts from Bangladesh and Ethiopia. Lepr. Rev..

[bib61] van Wijk R. (2021). Psychosocial burden of neglected tropical diseases in eastern Colombia: an explorative qualitative study in persons affected by leprosy, cutaneous leishmaniasis and Chagas disease – ERRATUM. Global Mental Health.

[bib62] Velink A. (2016). Former buruli ulcer patients' experiences and wishes may serve as a guide to further improve buruli ulcer management. PLoS Neglected Trop. Dis..

[bib63] WB (2016).

[bib64] WB (2021).

[bib65] WHO (2020).

[bib66] WHO (2022). WHO (World Health Organization).

[bib67] Wijerathna T. (2018). The economic impact of cutaneous leishmaniasis in Sri Lanka. BioMed Res. Int..

[bib68] Witter S. (2017). Minding the gaps: health financing, universal health coverage and gender. Health Pol. Plann..

[bib69] Yakupu A. (2023). The burden of skin and subcutaneous diseases: findings from the global burden of disease study 2019. Front. Public Health.

