# Supplementary material for: The role of economic factors in shaping and constituting the household burden of neglected tropical diseases of the skin: Qualitative findings from Ghana and Ethiopia
Source: Soc Sci Med. 2024 Sep;356:117094. doi: 10.1016/j.socscimed.2024.117094 (PMC11370647; doi:10.1016/j.socscimed.2024.117094)
Supplement: Multimedia component 1 [file mmc1.docx]

#

**Consolidated criteria for reporting qualitative studies (COREQ): 32-item checklist**

Framework from: Tong A, Sainsbury P, Craig J. Consolidated criteria for reporting qualitative research (COREQ): a 32-item checklist for interviews and focus groups. *International Journal for Quality in Health Care*. 2007. Volume 19, Number 6: pp. 349 – 357

| **No. Item** | **Guide questions/description** | **Response** |
| --- | --- | --- |
| **Domain 1: Research team and reﬂexivity** | | |
| *Personal Characteristics* |  |  |
| 1. Interviewer/facilitator | Which author/s conducted the interview or focus group? | Hailemichael Y, Alemu AY, Teklu C, Kebebew G and five trained research assistants with continous support from a large team from Armauer Hansen Research Institute, Addis Ababa University, and London School of Hygiene and Tropical Medicine.  In Ghana interviews were conducted by Daniel Otchere, Edmond Ocloo, Lucy Owusu and Ruth Tuwor, with support from senior researchers |
| 2. Credentials | What were the researcher’s credentials? E.g. PhD, MD | Interview team consisted of nine people in Ethiopia and 4 people in Ghana with a variety of professionals including one post-Doctoral fellow, one PhD student, two master’s degree holders, two master’s students and three first degree qualification |
| 3. Occupation | What was their occupation at the time of the study? | University lecturers, students and researchers. |
| 4. Gender | Was the researcher male or female? | Male and female. |
| 5. Experience and training | What experience or training did the researcher have? | Interviewers received qualitative research methods and analysis training through their academic educational training programmes. They were also trained on qualitative methods with support of the Skin Health Africa Research Program and CI (co-investigators) of this study. |
| *Relationship with participants* |  |  |
| 6. Relationship established | Was a relationship established prior to study commencement? | Before the start of data colleciton key community influential leaders were engaged within the respective communities selected for the research. During the forum, discussions focused on the purpose of the research, expectations from local leaders to facilitate community entry and the community as providers of evidnece as well as implicaions of the study outcome. At health facility level, key management members were oriented about the research, expectations and implications |
| 7. Participant knowledge of the interviewer | What did the participants know about the researcher? e.g. personal goals, reasons for doing the research | General research objectives were clearly explained, as well as the role of the interviewer and note taker |
| 8. Interviewer characteristics | What characteristics were reported about the interviewer/facilitator? e.g. Bias, assumptions, reasons and interests in the research topic | The research team that conducted interviews and FGDs were academic staff from research and academic institutions, with support from overseas collaborators. None of the field team members originally came from the study district but were all native speakers of the main local language (Amharic in Ethiopia and Twi in Ghana).  Interviewers and FGD moderators spoke Amharic, Twi and English languages and all interviews were conducted in a language of the respondents (Amharic/Twi).  Female research assistants were available in case gender preference is requested by the participants.  Interviewers consisted of people from a variety of disciplinary backgrounds, including social science, health economics and public health researchers, but none of them had prior experiences of working on skin NTDs. However, the senior authors of this study had immense research experience in CL, BU and leprosy. |
| **Domain 2: study design** | | |
| *Theoretical framework* |  |  |
| 9. Methodological orientation and Theory | What methodological orientation was stated to underpin the study? e.g. grounded theory, discourse analysis, ethnography, phenomenology, content analysis | A Conceptual framework was developed to guide the thematic analysis that was conducted. |
| *Participant selection* |  |  |
| 10. Sampling | How were participants selected? e.g., purposive, convenience, consecutive, snowball | In-depth interviews: We engaged community leaders (kebele chiefs, traditional healers, opinion leaders, religious leaders), community members, health extension workers and health center managers.  To identify affected individuals the line list of cases obtained at the health facilities was used and this was complemented by the snowball sampling technique,  FGDs: The FGD participants were recruited purposively based on the maximum variation sampling. |
| 11. Method of approach | How were participants approached? e.g. face-to-face, telephone, mail, email | All participants were approached face-to-face |
| 12. Sample size | How many participants were in the study? | 14 FGDs with 6-8 participants per group, 50 IDIs with leprosy and CL affected people and caregiver, health workers and community members |
| 13. Non-participation | How many people refused to participate or dropped out? Reasons? | None |
| *Setting* |  |  |
| 14. Setting of data collection | Where was the data collected? e.g. home, clinic, workplace | FGD sessions were conducted in communal space (in school compound or health center compound or open field under a shed), while IDIs were conducted in the participants home or place of work but in private space. |
| 15. Presence of non-participants | Was anyone else present besides the participants and researchers? | No non-participant was present during the FGD sessions. However, caregivers were sometimes present and invited to provide additional information during IDIs with children. |
| 16. Description of sample | What are the important characteristics of the sample? e.g. demographic data, date | Both gender (M and F), people with and without formal education and age range of 13 to 90 years participated in interviews at community level. Additionally, health workers who are involved in skin NTDs at district, level participated. The duration of the data collection was from March- June 2021. |
| *Data collection* |  |  |
| 17. Interview guide | Were questions, prompts, guides provided by the authors? Was it pilot tested? | Qualitative interview topic guides were developed for IDIs and FGDs. The IDI topic guide was developed with due account of participants’ age. All guides were translated to the local language. These guides are available on request. |
| 18. Repeat interviews | Were repeat interviews carried out? If yes, how many? | No. Most interviews with BU, CL and leprosy affected people, community members were single, but one interview with affected people with leprosy involved multiple interactions. |
| 19. Audio/visual recording | Did the research use audio or visual recording to collect the data? | Interviews and FGDs were audio recorded with participant consent and transcribed and translated into English for analysis. However, one participant did not agree to be recorded recording. Notes were therefore taken. |
| 20. Field notes | Were ﬁeld notes made during and/or after the interview or focus group? | Yes. Scribbles were expanded to field notes after the interviews/FGDs while full transcription and translation of voice records started after data collection is completed mid-way of the entire process.FGDs were facilitated by two researchers, one who guided the discussion with the other documenting field notes. Notes were also made during and after interviews |
| 21. Duration | What was the duration of the interviews or focus group? | For FGDs 60 minutes.  50 minutes for IDIs |
| 22. Data saturation | Was data saturation discussed? | Yes. The redundancy of information across the themes was used to declare data saturation. |
| 23. Transcripts returned | Were transcripts returned to participants for comment and/or correction? | No |
| **Domain 3: analysis and ﬁndings** | | |
| *Data analysis* |  |  |
| 24. Number of data coders | How many data coders coded the data? | Four data coders coded the data in Ethiopia. In Ghana, coding was done by two individuals. |
| 25. Description of the coding tree | Did authors provide a description of the coding tree? | Yes, a code book was developed to provide description of the coding elements |
| 26. Derivation of themes | Were themes identiﬁed in advance or derived from the data? | Partly identified in advance and partly derived from the data (Themes were mainly identified in line with the aim and objectives of the study before the data collection, however during data analysis, emerging themes were added and explored after reading the transcripts, and this informed revision of the codebook) |
| 27. Software | What software, if applicable, was used to manage the data? | MAXQDA plus 2020 |
| 28. Participant checking | Did participants provide feedback on the ﬁndings? | Key findings were presented to community representatives, NTDs program managers as well as BU, CL and leprosy affected people. The dissemination and co-creation workshops were held at community level and national level. |
| *Reporting* |  |  |
| 29. Quotations presented | Were participant quotations presented to illustrate the themes/ﬁndings? Was each quotation identiﬁed? e.g. participant number | Yes, with participants ID to make it anonymous |
| 30. Data and ﬁndings consistent | Was there consistency between the data presented and the ﬁndings? | Yes. |
| 31. Clarity of major themes | Were major themes clearly presented in the ﬁndings? | Yes. |
| 32. Clarity of minor themes | Is there a description of diverse cases or discussion of minor themes? | The discussion focuses on the key-findings, particularly on the economic impact of skin NTDs and the coping strategies implemented by household to mitigate the burden. |
